# Pearl Millet Cover Crop Extract Inhibits the Development of the Weed *Ipomoea grandifolia* by Inducing Oxidative Stress in Primary Roots and Affecting Photosynthesis Efficiency

**DOI:** 10.3390/plants14020222

**Published:** 2025-01-15

**Authors:** Gislaine Cristiane Mantovanelli, Adriano Antônio Silva, Letycia Lopes Ricardo, Fernanda Lima Kagami, Jéssica Dario de Almeida, Mauro Cezar Barbosa, Márcio Shigueaki Mito, Isabela de Carvalho Contesoto, Paulo Vinicius Moreira da Costa Menezes, Gabriel Felipe Stulp, Beatriz Pereira Moreno, Francielli Alana Pereira Valeze, Rubem Silvério de Oliveira Junior, Debora Cristina Baldoqui, Emy Luiza Ishii Iwamoto

**Affiliations:** 1 Laboratory of Biological Oxidations, Department of Biochemistry, State University of Maringa, Maringa 87020-900, PR, Brazil; gcmantovanelli2@uem.br (G.C.M.); fernandakagami@gmail.com (F.L.K.); jessicadario.a@gmail.com (J.D.d.A.); mcezarbarbosa@hotmail.com (M.C.B.); msmito2@uem.br (M.S.M.); isabelacontesoto@gmail.com (I.d.C.C.); pvmcmenezes2@uem.br (P.V.M.d.C.M.); gabriel_stiilp@hotmail.com (G.F.S.); 2 Federal University of Southern Frontier, Realeza Campus, Realeza 85770-000, PR, Brazil; adriano.silva@uffs.edu.br; 3 Faculty of Biopark, Toledo 85919-899, PR, Brazil; lelrfiorucci@gmail.com; 4 Laboratory of Synthesis and Natural Products, Department of Chemistry, State University of Maringa, Maringa 87020-900, PR, Brazil; pereira_moreno@hotmail.com (B.P.M.); alanafranhp@gmail.com (F.A.P.V.); dcbaldoqui@uem.br (D.C.B.); 5 Department of Agronomy, State University of Maringa, Maringa 87020-900, PR, Brazil; rsojunior@uem.br

**Keywords:** *Pennisetum glaucum*, crop protection, mitochondria, respiration, proline, weed management

## Abstract

The cover crop *Pennisetum glaucum* (L.) R.Br. (pearl millet) reduces the emergence of weed species in the field through a mechanism that is not fully known. The identification of the allelopathic activity of pearl millet can contribute to the development of no-tillage techniques to produce crops without or with low doses of herbicides. This issue was investigated by testing the effects of extracts from the aerial parts of pearl millet on the germination and growth of the weeds *Bidens pilosa* L., *Euphorbia heterophylla* L., and *Ipomoea grandifolia* (Dammer) O’Donell under laboratory conditions. The ethyl acetate fraction (EAF) at a concentration of 2000 µg mL^−1^ was inactive on *Bidens pilosa*; it inhibited root length (−72%) and seedling fresh weight (−41%) of *E. heterophylla*, and in *I. grandifolia* the length of primary root and aerial parts and the fresh and dry weight of seedlings were reduced by 63%, 32%, 25%, and 12%, respectively. In roots of *I. grandifolia* seedlings, at the initial development stage, EAF induced oxidative stress and increased electrolyte leakage. At the juvenile vegetative stage, a lower concentration of EAF (250 µg mL^−1^) induced a stimulus in seedling growth (+60% in root length and +23% in aerial parts length) that was associated with increased photosynthetic efficiency. However, at higher concentrations (1000 µg mL^−1^), it induced the opposite effects, inhibiting the growth of root (−41%) and aerial parts (−25%), with reduced superoxide dismutase activity and photosynthetic efficiency. The stilbenoid pallidol was identified as the main compound in EAF. The allelopathic activity of pearl millet may be attributed, at least in part, to the impairment of energy metabolism and the induction of oxidative stress in weed seedlings, with pallidol possibly involved in this action. Such findings demonstrated that the application of the EAF extract from pearl millet can be a natural and renewable alternative tool for weed control.

## 1. Introduction

Cover crops are used in sustainable agriculture, combining soil cover as a live crop or mulch and diversification of crop rotation [1]. These systems have several benefits, such as the recycling of nutrients through the gradual decomposition of organic waste [2], the improvement of soil hydrology by controlling soil infiltration, drainage and aeration, and weed control [3]. There are many examples of cover crops that are effective in suppressing weeds, including sorghum, wheat, sunflower, ryegrass, alfalfa, mustard, and red clover [4,5]. It has also been demonstrated that allelopathic plant extracts can be used to control weeds and reduce herbicides under field conditions [6]. Jamil et al. [7], for example, evaluated the herbicidal potential of sorghum aqueous extract alone and in combination with aqueous extracts of other allelopathic plants: eucalyptus, sesame, sunflower, tobacco, and brassica, and demonstrated that the application of sorghum and sunflower extracts is more effective than other combinations in reducing the growth of wild oat (*Avena fatua*) and canary grass (*Phalaris minor*).

Pearl millet, *Pennisetum glaucum* (L.) R.Br., has expanded in Brazil as one of the main cover crop alternatives [8] due to its good adaptability to the diversity of environments and climate [9]. In a study with *Zea mays* L., *Urochloa ruziziensis* (R.Germ. & Evrard) Crins, or *P. glaucum* cultivated in succession to soybean (*Glycine max* (L.) Merr.), de Souza et al. [10] showed that the soybean/*P. glaucum* succession produces greater biomass, with reduction in soil density and increase in total porosity in the surface layer, important factors in improving crop productivity in the no-tillage system. Branco et al. [11] reported that the organic mulch of *P. glaucum* stood out in reducing weed biomass and increasing tomato and broccoli productivity yield when compared with other cover crop species (*Crotalaria juncea* L., *Canavalia ensiformis* (L.), and *Sorghum bicolor* L. Moreover, *P. glaucum* has demonstrated reduction of the emergence of weed species, including *U. brizantha* (Hochst. ex A. Rich.) Stapf, *Medicago sativa* L., *Trianthema portulacastrum* L., and *Amaranthus* spp. [12,13,14,15,16].

Most studies on the effects of cover crops on weeds consider only the physical effect of the straw on the soil [17], the shading caused by vegetation cover, and the competition for spaces and nutrients, but it has been demonstrated that the release of chemical compounds from plant residue exerts great influence on weed emergence [18]. Actually, in our previous study, we found evidence that pearl millet straw reduces weed emergence by chemical effects under field-like conditions [19]: when *Euphorbia heterophylla* L. and *Bidens pilosa* L. seeds were sown in pots covered with millet straw (4 to 8 t ha^−1^ dry mass), a significant reduction in weed emergence was found only in pots irrigated by surface water supply, i.e., when water percolated through the straw deposited on the soil surface; when water was supplied by capillarity without passing through the straw layers, no inhibition was observed, indicating that the inhibitory effects are more chemical than physical.

In pearl millet grains, chemical substances, such as coumarins, steroids, flavonoids, flavanone, tannins, phenolic acids, sugars, phospholipids, fatty acid, and carotenoids, have been identified [20,21,22]. Many of these compounds are known to exert an allelopathic effect, interfering with mitochondrial energy metabolism [23]. Plants depend strictly on adenosine 5′-diphosphate (ADP) phosphorylation to germinate and grow. During germination and initial seedling development, this process depends exclusively on mitochondrial metabolism, and after the development of leaves, photosynthesis begins to contribute to both adenosine 5′-triphosphate (ATP) and nutrient supply to support plant development. Both mitochondria and chloroplasts are also the main sites of reactive oxygen species (ROS) generation, which are neutralized by the antioxidant defense systems under normal conditions [24,25]. A perturbation of these processes by exogenous chemical substances may affect the emergence and growth of weeds in the field [23].

To examine whether the suppressive effect of *P. glaucum* on weeds involves the chemicals released by its straw and their possible modes of action, in the present work, soluble chemical compounds extracted from the aerial parts of pearl millet were tested on the weed species *B. pilosa*, *E. heterophylla,* and *Ipomoea grandifolia* (Dammer) O’Donell. The hypothesis of interference in energy metabolism was investigated in the most sensitive weed *I. grandifolia* at two stages of weed development: at germination and initial seedling growth by measuring mitochondrial energy metabolism, and at the juvenile vegetative stage by evaluating photosynthetic efficiency. In both stages, the correlation between mitochondrial and photosynthetic functions and oxidative stress conditions was also evaluated.

## 2. Materials and Methods

### 2.1. Plant Materials

Pearl millet was cultivated at the Experimental Farm of the State University of Maringa, Iguatemi, Parana State, Brazil (S 23°21′21.9″; W 52°04′18.9″). The soil physicochemical properties were as follows: pH (6.9), sand (86%), silt (3%), clay (11%), and organic carbon (1.04% of organic matter). A voucher specimen was deposited at the herbarium of the State University of Maringa (HUEM 29323). The aerial parts were collected before flowering, and the resulting straw was oven-dried at 40 °C and chopped into small pieces.

### 2.2. Extraction and Isolation of Constituents of Pearl Millet

The dried straw of the aerial parts of pearl millet (650 g) was powdered and exhaustively extracted with methanol at room temperature. Vacuum concentration at 33–35 °C yielded the crude methanol extract (25.8 g), which was suspended in 1.0 L of methanol/water (3:1) and successively partitioned with 3 × 150 mL hexane, dichloromethane, ethyl acetate, and butanol. The solvents were removed under reduced pressure to yield the hexane (8.8 g), dichloromethane (1.7 g), ethyl acetate (1.6 g), butanol (3.2 g), and aqueous methanol (9.8 g) fractions (Figure S1). In this work, the ethyl acetate (EAF) and butanol fractions (ButF) were used.

### 2.3. Germination and Initial Growth of Weeds

Seeds of *Bidens pilosa*, *Euphorbia heterophylla*, and *Ipomoea grandifolia* were purchased from a commercial supplier (Cosmos Agrícola Produtos e Serviços Rurais Ltd.a., São Paulo, Brazil). The seeds of *B. pilosa* were soaked in 1% sodium hypochlorite for 5 min and then washed with distilled water. *E. heterophylla* seeds were washed with distilled water, and *I. grandifolia* seeds were scarified with sulfuric acid for 45 min to break seed dormancy and then washed with distilled water [26]. The seeds were placed on double sheets of germination paper in an acrylic germination box (110 mm × 110 mm × 50 mm) and moistened with 10 mL of distilled water (control) or with solutions of the ethyl acetate fraction (EAF) at concentrations of 500, 1000, or 2000 µg mL^−1^ and the butanol fraction (ButF) at a concentration of 2000 µg mL^−1^. These solutions were prepared by weighing the fractions obtained after removal of the solvent (Section 2.2) and dissolving them with distilled water. The pH was then adjusted to 6.0. This concentration range was defined in previous tests, revealing the concentrations in which the extracts exerted dose-dependent and near-maximal effects. Each treatment, including the controls, was replicated five times, and each replicate consisted of fifty seeds distributed over the sheets. The boxes were randomly placed in a germination chamber (photon flux density of approximately 230 μmol m^−2^ s^−1^). *B. pilosa* was maintained at a photoperiod of 8/16 h (light/dark) at 30 °C and 20 °C, respectively. *E. heterophylla* was maintained at a constant temperature of 25 °C with a 12 h photoperiod (light/dark), and *I. grandifolia* was maintained at a constant temperature of 30 °C with a 12 h photoperiod (light/dark). The germinated seeds were counted daily for 96 h, and after this time the aerial parts and the primary roots of the seedlings were excised for measurement of their lengths and fresh weights. The dry weight of seedlings was calculated after incubation of seedlings at 60 °C for 48 h.

The mean germination time ($\bar{t}$ = mean germination time), the speed of germination (S), and the speed of accumulated germination (AS) were calculated according to Chiapusio et al. [27].

### 2.4. Vegetative Plant Growth

For evaluation of *I. grandifolia* seedlings at the juvenile vegetative stage of development, the seeds were placed in 380 mL pots (upper diameter of 93 mm, lower diameter of 59 mm, and height of 122 mm) containing perlite (12 g/pot) and cultivated in a germination chamber for 30 days with a 12 h photoperiod (photon flux density of approximately 600 μmol m^−2^ s^−1^) at 25–30 °C. The plants were watered daily with 15 mL of Hoagland nutrient solution (control) or the EAF dissolved in Hoagland nutrient solution at a final concentration of 250, 500, or 1000 µg mL^−1^. On the 30th day of cultivation, the plants were removed, and the roots, stems, and leaves were excised to determine their lengths and fresh weights. The dry weights were estimated after oven-drying five plants per treatment at 60 °C. The leaf area was determined using a Li-3100C leaf area meter (LI-COR, São Paulo, Brazil).

### 2.5. Respiratory Activity Measurement of Primary Roots

The oxygen uptake by root apexes of *I. grandifolia* seedlings was measured in primary roots of seedlings grown for 96 h, polarographically at 25 °C with a Clark-type electrode (Probe Oxygen sensor 5331, Yellow Springs, OH, USA) positioned in a closed plexiglass chamber [28]. The primary roots were cut into 2–5 mm long segments (measured from the apex), and, after weighing, the samples were immediately placed into the chamber with 2.0 mL of a nutrient solution (pH 5.8) containing 2.0 mM Ca(NO_3_)_2_, 2.0 mM KNO_3_, 0.43 mM NH_4_Cl, 0.75 mM MgSO_4_, and 20 µM NaH_2_PO_4_. Potassium cyanide (KCN) at a final concentration of 200 µM was added to the reaction medium to estimate the contribution of mitochondrial cytochrome oxidase (COX; potassium cyanide-sensitive (KCN-sensitive) respiration) and alternative oxidase (AOX) plus the extramitochondrial oxidases (potassium cyanide-insensitive (KCN-insensitive) respiration) to the overall O_2_ uptake [29]. Each treatment, including the control, was replicated five times.

### 2.6. Determination of Malondialdehyde Content, Free Proline Content, and Electrolyte Leakage in Primary Roots of Ipomoea grandifolia Seedlings

Lipid peroxidation was measured as malondialdehyde (MDA) content using a thiobarbituric acid (TBA) reaction [30]. Approximately 0.6 g of primary roots excised from seedlings grown for 96 h was transferred to a mortar with 3 mL of cold (4 °C) 96% (*v*/*v*) ethanol. An equal volume of 10% (*v*/*v*) trichloroacetic acid (TCA) containing 0.5% (*w*/*v*) TBA was added to the homogenate and heated at 95 °C for 30 min. The reaction was stopped in an ice bath and centrifuged at 10,000 g for 10 min. The absorbance of the supernatant was read at 532 nm (ɛ = 155 mM^−1^ cm^−1^), and non-specific absorbance at 600 nm was subtracted [30]. Each treatment was replicated four to seven times.

Membrane integrity was determined by electrolyte leakage from the primary roots of *I. grandifolia* by using a conductivity meter. Primary roots (0.2–0.3 g) from seedlings were excised, weighed, and placed into 30 mL of deionized water at room temperature. The roots were incubated for 4 h, and then the conductivity was measured (Tecnal TEC-4MP, São Paulo, Brazil). Afterward, the roots were transferred to another beaker with 30 mL of deionized water and boiled for 15 min. At room temperature, conductivity was measured again. The total conductivity was calculated as the ratio between the measures. The results were expressed in µS cm^−1^ g^−1^ fresh weight. The conductivity cell used was number 1, and the standard cell was 146.9 µS cm^−1^.

The levels of proline were analyzed using the acid ninhydrin method [31]. Approximately 0.5 g of tissue was homogenized in 6 mL of 3% sulfosalicylic acid (*w*/*v*) and filtered on paper, and 2 mL of the extract was mixed with 2 mL of 6 M ninhydrin acid solution and 2 mL of glacial acetic acid. The samples were incubated at 100 °C for 1 h and then placed on ice. Four milliliters of toluene were added to the solution, and stirred for 15 s for proline extraction. After resting, the less dense part (chromophore) was aspirated and read in a spectrophotometer at 520 nm. The absorbances were compared to the standard proline curve (0 to 100 μg mL^−1^), and the results were expressed in μmol g^−1^ fresh weight.

### 2.7. Determination of Antioxidant Enzyme Activity

The primary roots (0.1 g) of seedlings grown for 96 h or leaves (0.1 g) from seedlings grown for 30 days were removed from treated and untreated seedlings, transferred to a mortar, and thoroughly mixed with 2.5 mL of 0.1 M phosphate buffer (pH 6.8) containing 0.1 mM ethylene diamine tetraacetic acid (EDTA) and 0.1% polyvinylpyrrolidone (PVP). Extracts were centrifuged for 30 min at 7400 g at 4 °C, and the supernatant was used as the enzyme source. Superoxide dismutase (SOD, EC 1.15.1.6) activity was measured by the inhibition of the photochemical reaction of nitro blue tetrazolium (NBT) [32]. The medium contained 50 mM phosphate buffer (pH 7.8), 13 mM methionine, 75 µM NBT, 2 µM riboflavin, 0.1 mM EDTA, and 0.1–0.2 mg protein of enzyme extract. One unit of SOD activity (U) was defined as the amount of enzyme required to cause 50% inhibition of the NBT photochemical reaction at 560 nm. Catalase (CAT, EC 1.11.1.6) activity was measured in a medium containing 100 mM phosphate buffer (pH 7.0), 15 mM hydrogen peroxide (H_2_O_2_), and 0.1–0.2 mg protein of enzyme extract. The consumption of H_2_O_2_ was monitored at 240 nm (ε = 39.4 M^−1^ cm^−1^) [33]. Peroxidase (POD, EC 1.11.1.7) activity was measured in a medium containing 100 mM phosphate buffer (pH 7.0), 20 mM pyrogallol, 10 mM H_2_O_2_, and 0.1–0.2 mg protein of enzyme extract. The peroxidase activity was calculated by determining the amount of purpurogallin formed at 420 nm (ε = 2.8 mM^−1^ cm^−1^) [34]. Ascorbate peroxidase (APX, EC 1.11.1.11) activity was measured in a medium containing 50 mM phosphate buffer (pH 7.0), 0.5 mM ascorbate, 0.1 mM H_2_O_2_, and 0.1–0.2 mg protein of enzyme extract. Ascorbate oxidation was monitored at 290 nm (ε = 2.8 mM^−1^ cm^−1^) [35]. Glutathione reductase (GR, EC 1.8.1.7) activity was measured in a medium containing 100 mM phosphate buffer (pH 7.5), 0.1 mM EDTA, 1 mM oxidized glutathione (GSSG), 0.1 mM nicotinamide adenine dinucleotide phosphate (NADPH), and 0.1–0.2 mg protein of enzyme extract. The rate of NADPH oxidation was monitored at 340 nm (ε = 6.22 mM^−1^ cm^−1^) [36]. The concentration of total protein in the enzyme extracts was determined using the Bradford method [37]. Bovine serum albumin was used as the standard.

### 2.8. Gas Exchange and Relative Chlorophyll Content

Seedlings grown in the absence or presence of 250, 500, and 1000 μg mL^−1^ of EAF for 30 days were subjected to fluorometric and gas exchange analysis by an infrared gas analyzer (IRGA; ADS Bio Scientific LC pro^+^, Hoddesdon, UK). The measurements were performed on fully expanded leaves under a photosynthetic photon flux density (PPFD) of 1200 μmol m^−2^ s^−1^ at 25 °C between 7:00 a.m. and 11:30 a.m. The following parameters were calculated: photosynthetic rate (A), stomatal conductance (g_s_), transpiration (E), intercellular CO_2_ concentration (Ci), A/Ci ratio, immediate carboxylation of the photosynthetic system [38], and water use efficiency (WUE) calculated as the ratio of A/E [39].

Relative chlorophyll content was assessed using a SPAD meter (SPAD-502, Minolta, Ramsey, NJ, USA). This portable device measures the difference between the transmittance of a red (650 nm) and an infrared (940 nm) light to generate a SPAD value (s). The transmittance at 650 is proportional to the concentration of chlorophyll a and b, and to the path length through the leaf. log_10_ transmittance at 940 nm (T940) is used to account for differences in path length. The equation for the SPAD-502 chlorophyll meter value (s) is

$$s=klog_{10}\left(\frac{T_{940}}{T_{650}}\right)$$

where *k* is the calibration coefficient [40]. For relative chlorophyll content analyses, the SPAD sensor was randomly placed in the leaf mesophyll tissue, avoiding the main veins. Two fully expanded younger leaves were measured per seedling and averaged to provide a single SPAD value

### 2.9. Analysis of EAF by HPLC-DAD (High-Performance Liquid Chromatography-Diode Array Detector) and UHPLC-HRMS/MS (Ultra-High-Performance Liquid Chromatography (UHPLC)-High-Resolution Mass Spectrometry (HRMS)

HPLC exploratory analyses were performed on a Shimadzu instrument (Mod. Prominence, Kyoto, Japan) with two LC-20AR pumps, degasser DGU-20ASR, diode array detector (DAD) SPD-M20A model, and automatic injection system SIL-10AF, and equipped with a Shim-pack PREP-ODS (250 mm × 20 mm; 15 μm particle size) column. The mobile phase consisted of water (Milli-Q, Millipore) and acetonitrile (Merck, Millipore, St. Louis, MO, USA). The UHPLC analysis was performed in a Shimadzu Nexera X2 instrument, equipped with a CBM-20A system controller, two LC-30AD pumps, a CTO-30A column oven, and SIL-30AC autosampler. The mass spectra were recorded on a Bruker IMPACT II mass spectrometer (Billerica, MA, USA), with electrospray ionization source (ESI) in the positive ion mode, quadrupole time-of-flight (Q-TOF) analyzer and multichannel plate (MCP) detector. Experimental details are in the Supplementary Materials.

### 2.10. Statistical Analysis

**Figure 1:**
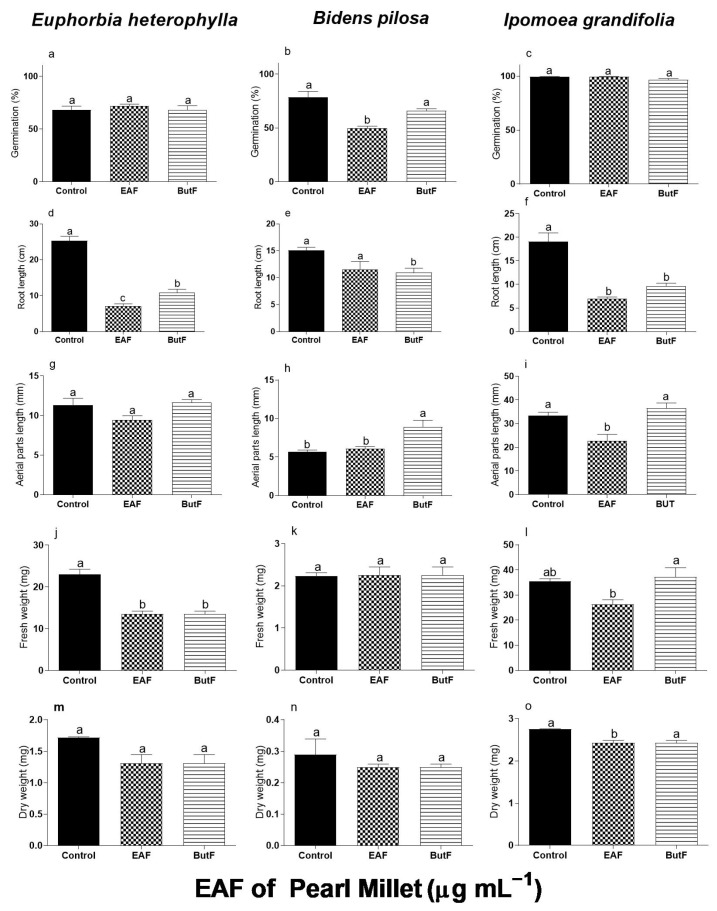
The effects of the EAF (ethyl acetate fraction) and ButF (butanolic fraction) of pearl millet on germination (%) (**a**,**b**,**c**), primary root length (**d**,**e**,**f**), aerial parts length (**g**,**h**,**i**), fresh weight (**j**,**k**,**l**), and dry weight of seedlings (**m**,**n**,**o**) of *Euphorbia heterophylla*, *Bidens pilosa*, and *Ipomoea grandifolia*. The seeds were incubated for 96 h in the presence of water (control), EAF, or ButF of pearl millet at 2000 µg mL^−1^ concentration. Each data point is the mean + SE (*n* = 5). Significant differences between means were identified using ANOVA with Tukey’s HSD test. Different letters indicate that the treatment means differed significantly at *p* ≤ 0.05.

**Figure 2:**
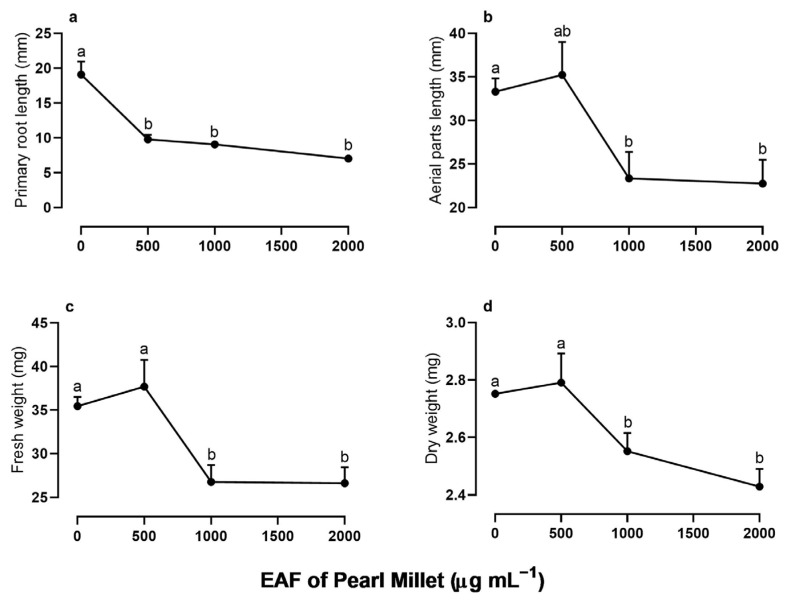
The effects of the EAF (ethyl acetate fraction) of pearl millet on the primary root length (**a**), aerial parts length (**b**), fresh weight (**c**), and dry weight (**d**) of *Ipomoea grandifolia*. The seeds were incubated for 96 h, in the presence of water (control) or the EAF of pearl millet (500–2000 µg mL^−1^). Each data point is the mean + SE (*n* = 5). Significant differences between means were identified using ANOVA with Tukey’s HSD test. Different letters indicate that means differed significantly at *p* ≤ 0.05.

**Figure 3:**
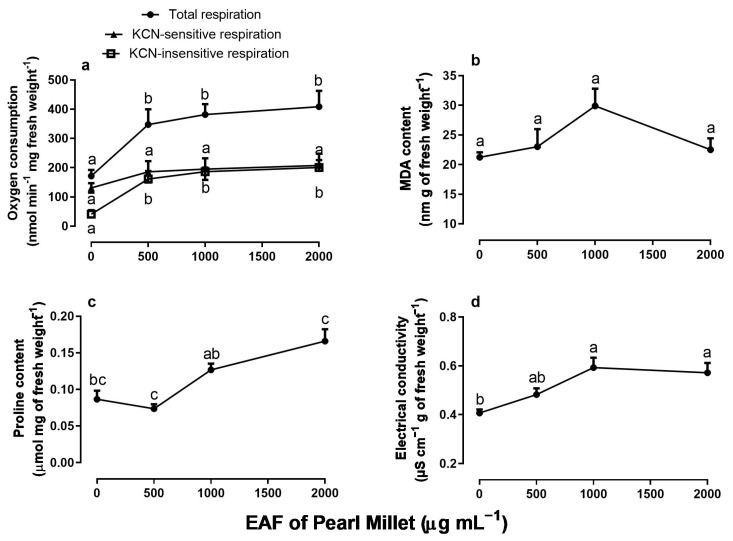
The effects of the EAF (ethyl acetate fraction) of pearl millet on total respiration (*n* = 5), potassium cyanide-insensitive (KCN-insensitive) respiration (*n* = 4), and potassium cyanide-sensitive (KCN-sensitive) respiration (*n* = 4–6) (**a**); MDA content (**b**) (*n* = 4–5); proline content (**c**) (*n* = 4–5); and electrical conductivity (**d**) (*n* = 4–5) of *Ipomoea grandifolia* roots from seedlings grown for 96 h in the presence of water (control) or the EAF of pearl millet (500–2000 µg mL^−1^). Each data point is the mean + SE. Significant differences between means were identified by ANOVA with Tukey’s HSD test. Different letters indicate that means differed significantly at *p* ≤ 0.05.

**Figure 4:**
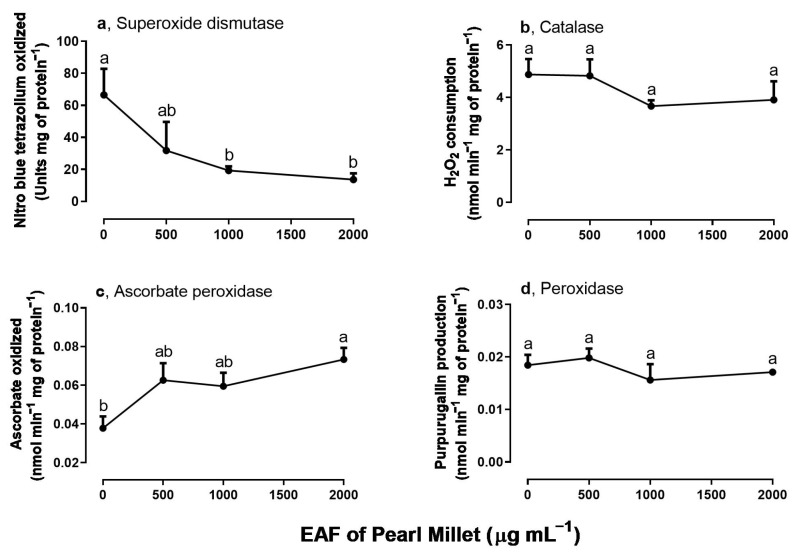
The effects of the EAF (ethyl acetate fraction) of pearl millet on the activities of superoxide dismutase (**a**) (*n* = 5), catalase (**b**) (*n* = 5), ascorbate peroxidase (**c**) (*n* = 7), and peroxidase (**d**) (*n* = 6) of *Ipomoea grandifolia* roots from seedlings grown for 96 h in the presence of water (control) or the EAF of pearl millet (500–2000 µg mL^−1^). Each data point is the mean + SE. Significant differences between means were identified by ANOVA with Tukey’s HSD test. Different letters indicate that means differed significantly at *p* ≤ 0.05.

**Figure 5:**
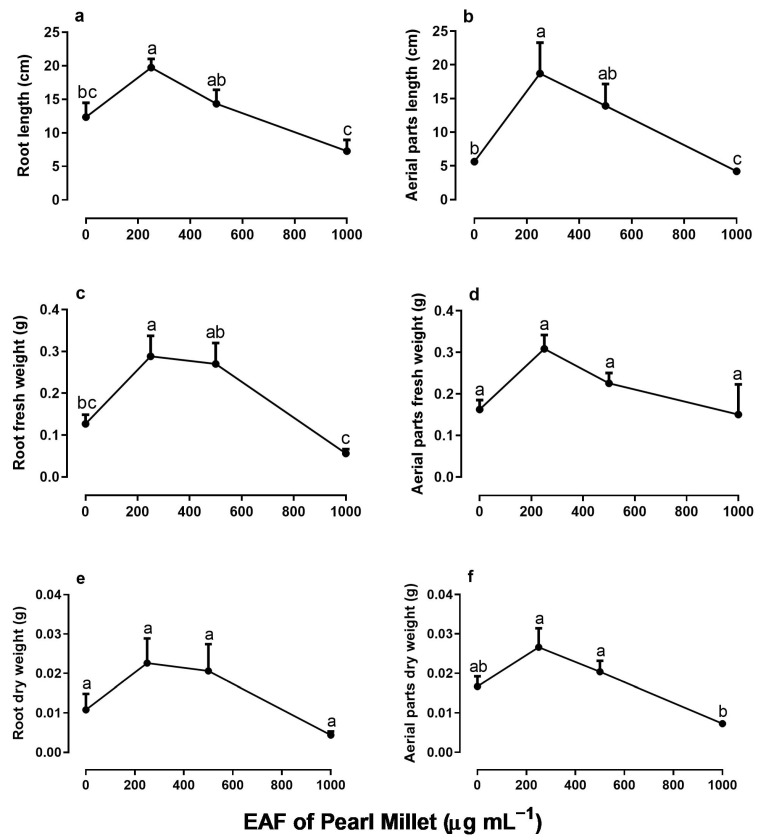
The effects of the EAF (ethyl acetate fraction) of pearl millet on the root length (**a**), aerial part length (**b**), root fresh weight (**c**), aerial part fresh weight (**d**), root dry weight (**e**), and aerial part dry weight (**f**) of *Ipomoea grandifolia*. The plants were grown for 30 days in the presence of water (control) or the EAF of pearl millet (250–1000 µg mL^−1^). Each data point is the mean + SE (*n* = 5). Significant differences between means were identified by ANOVA with Tukey’s HSD test. Different letters indicate that means differed significantly at *p* ≤ 0.05.

**Figure 6:**
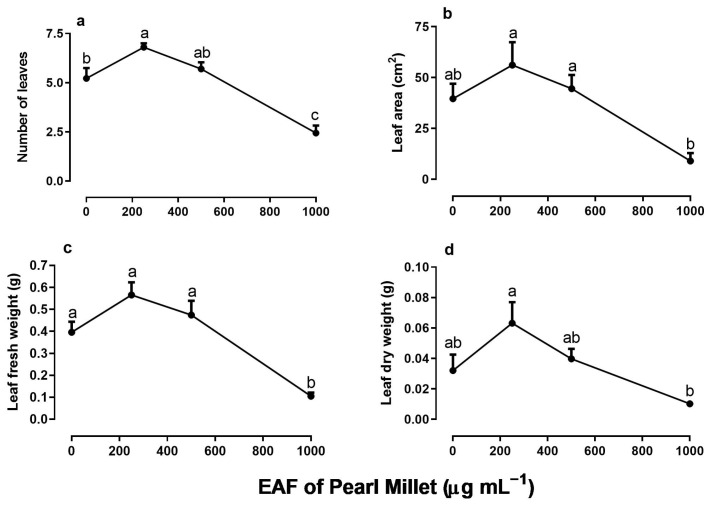
The effects of the EAF (ethyl acetate fraction) of pearl millet on the number of leaves (**a**), leaf area (**b**), leaf fresh weight (**c**), and leaf dry weight (**d**) of *Ipomoea grandifolia*. The plants were grown for 30 days in the presence of water (control) or the EAF of pearl millet (250–1000 µg mL^−1^). Each data point is the mean + SE (*n* = 5). Significant differences between means were identified by ANOVA with Tukey’s HSD test. Different letters indicate that means differed significantly at *p* ≤ 0.05.

**Figure 7:**
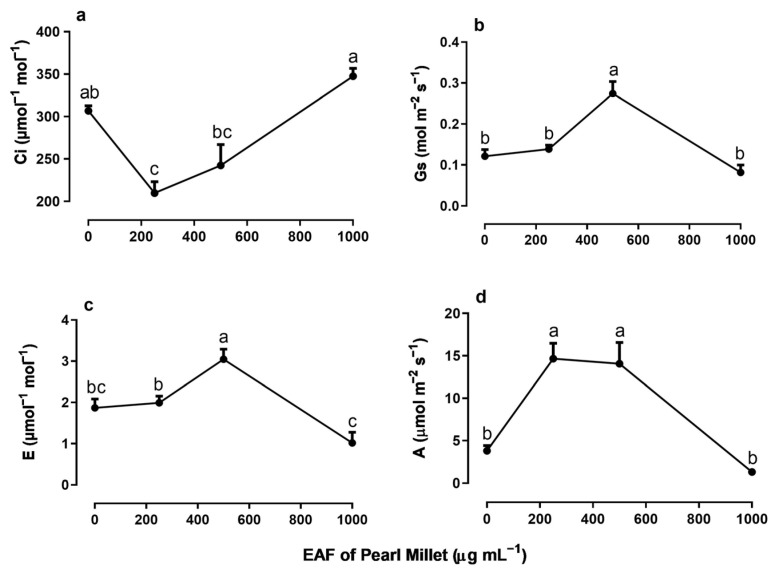
The effects of the EAF (ethyl acetate fraction) of pearl millet on the intercellular CO_2_ concentration, Ci (**a**), stomatal conductance, g_s_ (**b**), transpiration, E (**c**), and photosynthetic rate, A (**d**) of *Ipomoea grandifolia*. The plants were grown for 30 days in the presence of water (control) or the EAF of pearl millet (250–1000 µg mL^−1^). Each data point is the mean + SE (*n* = 5). Significant differences between means were identified by ANOVA with Tukey’s HSD test. Different letters indicate that means differed significantly at *p* ≤ 0.05.

**Figure 8:**
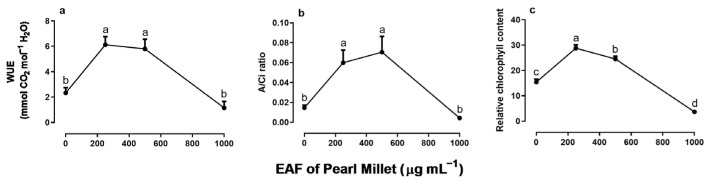
The effects of the EAF of pearl millet on the water use efficiency (WUE) (**a**), A/Ci ratio (**b**), and relative chlorophyll content (**c**) of *Ipomoea grandifolia*. The plants were grown for 30 days in the presence of water (control) or the EAF of pearl millet (250–1000 µg mL^−1^). Each data point is the mean + SE (*n* = 5). Significant differences between means were identified by ANOVA with Tukey’s HSD test. Different letters indicate that means differed significantly at *p* ≤ 0.05.

**Figure 9:**
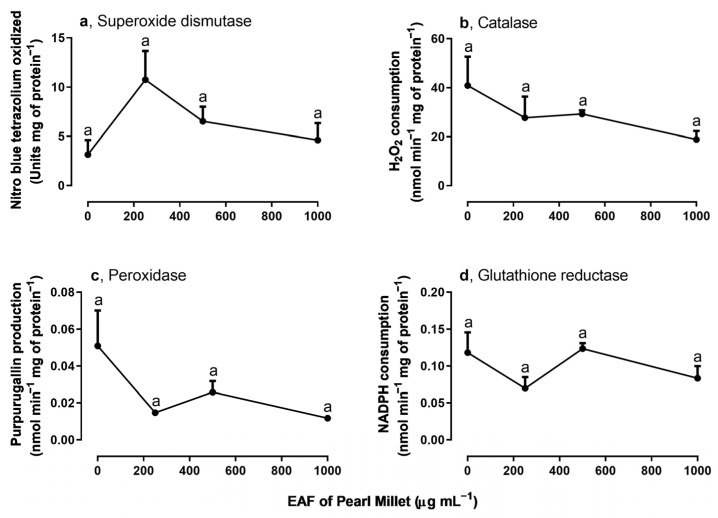
The effects of the EAF (ethyl acetate fraction) of pearl millet on the activities of superoxide dismutase (**a**) (*n* = 3–4), catalase (**b**) (*n* = 3–4), peroxidase (**c**) (*n* = 3–5), and glutathione reductase (**d**) (*n* = 4–5) of *Ipomoea grandifolia* leaves from seedlings grown for 30 days in the presence of water (control) or the EAF of pearl millet (250–1000 µg mL^−1^). Each data point is the mean + SE. Significant differences between means were identified by ANOVA with Tukey’s HSD test. Different letters indicate that means differed significantly at *p* ≤ 0.05.

The data presented in the graphs are expressed as mean ± standard error (S.E.) of independent preparations. All data were examined for normality using the Shapiro–Wilk test, which demonstrated a normal distribution of the data. Subsequently, the data were subjected to a one-way analysis of variance to compare the effects of EAF and ButF at a fixed concentration (Figure 1) and to compare the effects of different concentrations of EAF on the measured parameter (Figure 2, Figure 3, Figure 4, Figure 5, Figure 6, Figure 7, Figure 8 and Figure 9). Whenever a significant main effect was detected (*p* ≤ 0.05), additional comparisons were determined using Tukey’s honestly significant difference (HSD) test (*p* ≤ 0.05). The analytical process was performed using RStudio version 4.3.3 software.

### 2.11. Chemicals

The KCN, TBA, NBT, GSSG, and NADPH were purchased from Sigma Chemical Co. (St. Louis, MO, USA). The other reagents acquired were of the highest purity grade available.

## 3. Results

### 3.1. Effects of the EAF and ButF of Pearl Millet on Germination and Initial Growth of Euphorbia heterophylla, Bidens pilosa, and Ipomoea grandifolia

Figure 1 compares the effects of the EAF and ButF of pearl millet at a concentration of 2000 µg mL^−1^ on the germination and initial growth of *E. heterophylla*, *B. pilosa*, and *I. grandifolia*. The EAF and ButF did not alter the germination of *E. heterophylla* and *I. grandifolia*, but in *B. pilosa* the EAF reduced germination by 36.8% relative to untreated seeds. Furthermore, seeds treated with ButF germinated 24.8% more than seeds treated with EAF (Figure 1b). In contrast with this effect on germination, the growth of seedlings was altered by both fractions but with different sensitivities among the assayed species. *B. pilosa* was less sensitive, with a reduction in the primary root length (−28%) by ButF (Figure 1e). The treatment with ButF increased the length of the aerial parts by 45.6% compared to the length with EAF (Figure 1h). In *E. heterophylla*, both EAF and ButF decreased the root length (−72% and −75.5%, respectively, Figure 1g) and seedling fresh weight (−41% and −27%, respectively, Figure 1j). The roots of *E. heterophylla* treated with ButF were 13% longer than the roots treated with EAF (Figure 1b).

In *I. grandifolia*, a differential effect of EAF and ButF was observed. The ButF only reduced the length of the primary root (−49%) (Figure 1f), whereas the EAF caused inhibition in all biometric parameters of seedling development: primary root (−63%) (Figure 1f), aerial part length (−32%) (Figure 1i), and fresh (−26%) (Figure 1l) and dry weight (−12%) (Figure 1o) of seedlings. Based on these results, we selected the EAF of pearl millet to evaluate its mode of action on the most sensitive weed *I. grandifolia*.

In the initial stages of *I. grandifolia* development (96 h), the EAF, despite having little effect on germination indexes (Table S1), significantly altered the growth of the seedlings (Figure 2). There was a dose-dependent reduction in the length of the primary roots when compared with the untreated seedlings, with 44%, 52.5%, and 63% inhibition at EAF concentrations of 500, 1000, and 2000 µg mL^−1^, respectively (Figure 2a). The EAF also reduced the length of aerial parts and the fresh and dry biomass of the whole seedlings (Figure 2b–d). At the highest concentration of 2000 µg mL^−1^, these parameters were reduced by 46.5%, 33%, and 13%, respectively.

### 3.2. Effects of the EAF of Pearl Millet on Respiratory Activity of Root Apexes, Malondialdehyde Content, Free Proline Content, and Electrolyte Leakage of Ipomoea grandifolia

Measurements of oxygen consumption in root apexes revealed that EAF causes a stimulus in total respiration, reaching a 138% higher value relative to the control at the concentration of 2000 µg mL^−1^ (Figure 3a). The KCN-insensitive respiration represented 24% of the total respiration in untreated seedlings, and an increase of 46.3%, 47.7%, and 48.9% occurred when seedlings were grown in the presence of 500, 1000, and 2000 µg mL^−1^ of EAF, respectively. KCN-sensitive respiration was not modified (Figure 3a).

Figure 3 shows that EAF increased the content of MDA and proline in the primary root (Figure 3b,c). When compared with untreated seedlings, the increase in MDA was significant at 1000 µg mL^−1^ (+41%) and in proline at concentrations of 1000 (+46.5%) and 2000 µg mL^−1^ (+91%). The electrical conductivity of the roots also increased by 46% and 41% in the treatments with 1000 and 2000 µg mL^−1^, respectively (Figure 3d).

### 3.3. Effects of the EAF of Pearl Millet on the Activities of Antioxidant Enzymes of Ipomoea grandifolia Roots

Among the assayed antioxidant enzymes SOD, CAT, APX, and POD (Figure 4a–d), SOD and APX activities were altered by treatment with EAF. At the highest concentration of 2000 µg mL^−1^ SOD activity was reduced by 79.5% (Figure 4a), and APX activity was increased by 93.7% (Figure 4c).

### 3.4. Effects of the EAF of Pearl Millet on Biometric Parameters of Ipomoea grandifolia Grown for 30 Days

When seedlings were treated daily with EAF for 30 days, a double effect was observed in the growth of *I. grandifolia*. The lower concentration of 250 µg mL^−1^ induced a stimulus in seedling growth, but at higher concentrations an inhibitory effect began to prevail, so that the seedlings treated with 1000 µg mL^−1^ of EAF showed significantly lower growth than the control (Figure 5). At a concentration of 250 µg mL^−1^, the roots and aerial parts of seedlings, which were 60% and 232% higher than the control, respectively, were reduced to 41% and 25%, respectively, in the presence of 1000 µg mL^−1^ EAF (Figure 5a,b). The fresh and dry biomass of roots and aerial parts followed the same pattern of responses (Figure 5c–f).

In line with the increase in the biometric parameters of the aerial parts, the number of leaves increased in the treatment with 250 µg mL^−1^ of the EAF (+30%) and decreased at higher concentrations (Figure 6a). At the highest concentration of 1000 µg mL^−1^, the number of leaves was 54% lower than the control, and their mean area was 77% lower than that of the untreated seedlings (Figure 6b). The fresh and dry biomass of the leaf showed a pattern similar to the growth parameters: an increase in the concentration of 250 µg mL^−1^, followed by inhibition at higher concentrations (Figure 6c,d).

### 3.5. Effects of the EAF of Pearl Millet on Gas Exchange and Chlorophyll Content in Leaves of Ipomoea grandifolia Grown for 30 Days

The EAF at a concentration of 250 µg mL^−1^—a dose that stimulated the growth of seedlings—reduced the intercellular CO_2_ concentration (Ci) by 47%, without altering the stomatal conductance (g_s_) or transpiration (E) compared with the untreated seedlings (Figure 7a–c). Under the same conditions, values of net CO_2_ assimilation (A), water use efficiency (WUE), immediate carboxylation of the photosynthetic system (A/Ci ratio) and relative chlorophyll content increased by 287% 197%, 330%, and 87%, respectively (Figure 8a–c).

In plants treated with 500 µg mL^−1^ EAF, some changes not observed at 250 µg mL^−1^ were found. The most significant was an increase in g_s_ (+125%) and E (+63%) (Figure 7b,c). The Ci concentration remained lower than that of the control but slightly higher than that of plants treated with 250 µg mL^−1^ EAF (Figure 7a). The values of A, WUE, A/Ci ratio, and relative chlorophyll content were 271%, 148.5%, 400%, and 87% higher, respectively, than those of the controls, but the values were not significantly different from those of treatment with 250 µg mL^−1^ EAF.

In plants treated with the highest concentration of EAF (1000 µg mL^−1^), which caused a significant reduction in seedling growth, the pattern of the responses was the opposite of those found at the lower concentrations. The C_i_ increased by 12% (Figure 7a), and all other parameters that were stimulated at lower concentrations were reduced, reaching values similar to those of the control (Figure 7b–d and Figure 8a,b). Chlorophyll content was reduced by 80% (Figure 8c).

### 3.6. Effects of the EAF of Pearl Millet on the Activities of Antioxidant Enzymes in Leaves of Ipomoea grandifolia Grown for 30 Days

The evaluation of the activity of some antioxidant enzymes in the leaves of *I. grandifolia* revealed that the activity of catalase, peroxidase, and glutathione reductase was not modified (Figure 9b–d), but SOD activity was significantly altered by the EAF. A stimulus of 246% was observed in the treatment with 250 µg mL^−1^, but at higher concentrations the enzyme activity declined to values similar to those of the untreated seedlings (Figure 9a).

### 3.7. Identification of pallidol in the Ethyl Acetate Fraction of P. glaucum

**Figure 10:**
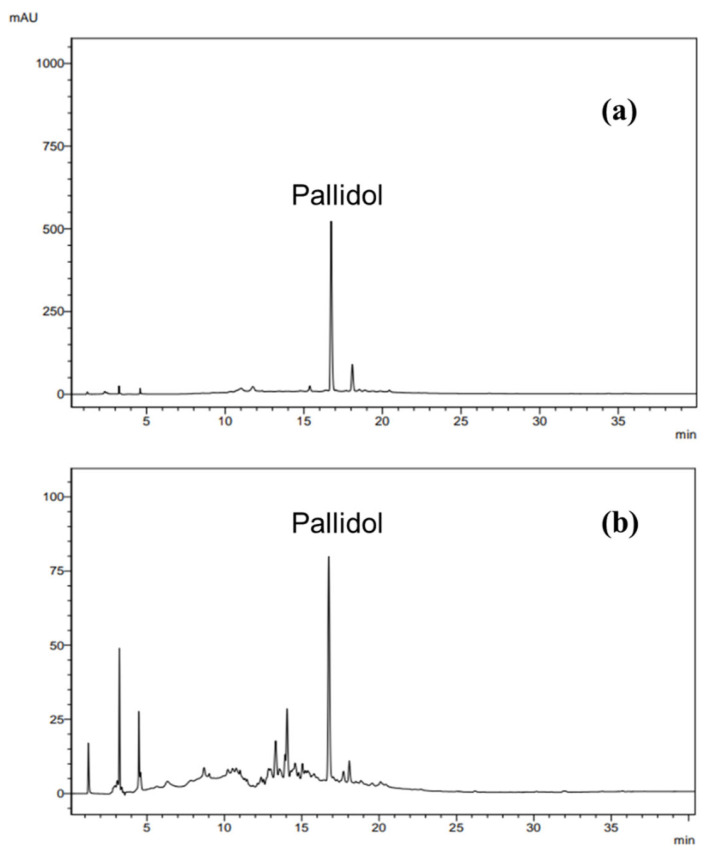
Chromatograms obtained by injection of pallidol (**a**) and ethyl acetate fraction (**b**). Analysis conditions: elution gradient, 5% A to 100% A (0–30 min) and 100% A (30–45 min); solvent A: acetonitrile; solvent B: water; flow rate 1.0 mL/min at λ = 282 nm.

**Figure 11:**
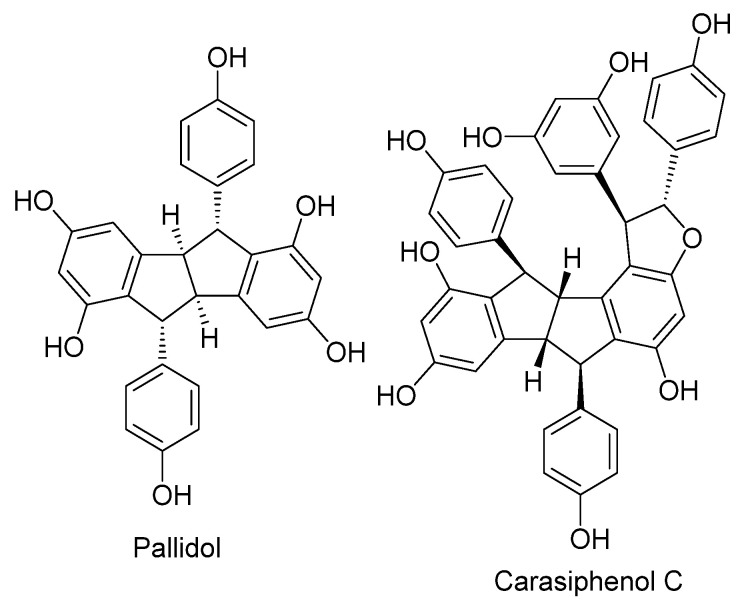
Chemical structures of pallidol and carasiphenol C.

Pallidol was identified in the EAF of *P. glaucum* by comparing its experimental NMR spectroscopic data with previously reported data [41]. HPLC analysis showed that pallidol is the major compound in the ethyl acetate fraction (Figure 10). To obtain the annotation of other resveratrol derivatives present in the ethyl acetate fraction of *P. glaucum*, this fraction was analyzed by UHPLC-HRMS/MS quadrupole time-of-flight (Q-TOF) in negative ionization mode (Figures S2 and S3, Table S2). This strategy allowed the putative identification of carasiphenol C in addition to pallidol (Figure 11).

## 4. Discussion

This work revealed that the aerial parts of *P. glaucum* contain soluble substances with allelopathic activity on the weeds *B. pilosa*, *E. heterophylla,* and *I. grandifolia*. The fractions obtained from extraction with ethyl acetate (EAF) and butanol (ButF) altered germination and seedling growth with different sensitivities among the species tested. The investigation of the mechanism of action of EAF in the most sensitive weed species *I. grandifolia* revealed that the effects of EAF from pearl millet depend on the stage of development of *I. grandifolia*. In the concentration range of 500 to 2000 µg mL^−1^, EAF inhibited the initial development of seedlings (96 h), but in plants in the juvenile vegetative stage of development (30 days), it exerted a double effect: stimulation at lower concentrations and inhibition at higher concentrations.

In its initial stages of growth, the embryo and seedling are basically heterotrophic. During seed imbibition, ATP generation depends on fermentative pathways, but as soon as the seed coat ruptures, the mitochondria of the embryonic tissues oxidize stored nutrients and generate ATP [23,42,43,44]. Mitochondrial metabolism is particularly active in root apexes, as the meristematic tissues have a high capacity for cell division, elongation, and/or differentiation [45]. Measurements of oxygen consumption in root apexes revealed that EAF causes a stimulus in total respiration. The total respiration of the root apexes represents the sum of all reactions that consume oxygen in the tissues. About 80–90% of cellular oxygen consumption is used to meet the energy needs of the cell through mitochondrial oxidative phosphorylation (via cytochrome oxidase, COX) [46]. The addition of the cytochrome oxidase inhibitor KCN (Figure 3a) revealed that there was no change in respiration linked to ADP phosphorylation via the COX pathway (KCN-sensitive respiration), indicating that the reduction in growth was not due to direct action on mitochondrial ATP generation. An increase in the KCN-insensitive respiration accounted for the increase in total respiration. Although our results do not allow us to distinguish which specific KCN-insensitive oxygenase was stimulated by the EAF, the mitochondrial alternative oxidase (AOX) and the extramitochondrial enzymes, especially lipoxygenases or NADPH oxidases, are generally activated in the condition of oxidative stress induced by excess ROS in tissues [47,48]. Activation of the monooxygenase system to metabolize EAF substances could also be considered [49]. All these assumptions still need to be confirmed in future studies.

Part of the increased oxygen consumption of root apexes may have been due to oxygen converted to superoxides (O_2_^•−^) and H_2_O_2_, which under normal conditions represent 2 to 3% of the oxygen used by mitochondria [50]. It remains to be measured whether ROS levels in root tissues are indeed elevated under EAF treatment, but evidence in favor of this possibility is the increase in MDA levels in roots. ROS can react with the polyunsaturated fatty acids of membrane phospholipids, leading to the peroxidation of fatty acid chains [51] and the formation of several secondary products, including MDA, a thiobarbiturate-reactive substance (TBARS) [52]. It is also likely that membrane lipid peroxidation contributed to the increase in ion leakage observed in *I. grandifolia* roots.

To modulate cellular ROS levels, plants contain soluble antioxidant compounds, such as ascorbic acid, vitamin E, and glutathione [53], and a battery of enzymes that can efficiently neutralize O_2_^•−^ radicals and H_2_O_2_, including SOD, CAT, POD, and enzymes of the thioredoxin system and the ascorbate peroxidase cycle [54]. The activities of SOD, CAT, POD, and APX were evaluated in primary roots during the initial phase of development, and significant inhibition by EAF was found in the activity of SOD. SOD catalyzes the dismutation of free O_2_^•−^ to form O_2_ and H_2_O_2_ [55]. Among the antioxidant enzymes, there was stimulation in APX activity, but not POD and catalase; thus, an accumulation of cellular superoxide was likely an early event in the induction of lipid peroxidation and oxidative stress by the EAF in the primary roots of *I. grandifolia.* A similar effect was reported by Zeng et al. [56] with the allelochemical secalonic acid F produced by the fungus *Aspergillus japonicus*. The authors observed a reduction of SOD associated with an increase in MDA in the weeds *B. pilosa* and *Echinochloa crus-galli* L. and in the plants *Sorghum bicolor* L. Moench and *Oryza sativa* L. Thus, an alteration in antioxidant enzymes makes plants vulnerable to oxidative damage [57].

Additional evidence that EAF induced oxidative stress was the increased proline content in the roots. Proline constitutes less than 5% of the total free amino acids in plant tissues under normal conditions, but under various forms of stress the proline concentration can reach 80% of the total amino acid pool [58]. Proline stabilizes proteins, preventing their denaturation [59]. In addition, there are reports that free proline neutralizes the hydroxyl radical [60,61], protecting the membrane and proteins from oxidative damage [62] and stabilizing the enzymes of the antioxidant system [63]. Despite this protective mechanism, cellular oxidative damage caused by EAF probably prevailed in *I. grandifolia* seedlings, as indicated by reduced SOD activity, increased MDA content, and increased root ion leakage. Thus, induction of oxidative stress seems to be a key event in the inhibitory action of EAF on *I. grandifolia* at its initial growth stage.

These effects of EAF can be attributed to pallidol, which was identified as the main compound of the fraction. Pallidol and carasiphenol C—also identified in EAF—are stilbenoid oligomers derived from resveratrol widely found in plants belonging to Vitaceae and Fabaceae [64,65,66,67]. In a previous study, Silva et al. [41] isolated pallidol and carasiphenol C from the ethyl acetate fraction of seed and root extracts of *Centrus echinatus* L. This species and *P. glaucum* are both grasses belonging to the Poaceae family. Antioxidant activity measured by scavenging superoxide anions produced in the xanthine/xanthine oxidase system and DPPH radicals showed that pallidol is a weak antioxidant compared to resveratrol and quercetin [68].

Pallidol is concentrated in EAF due to its amphipathic characteristics [69], a property that favors interactions with the lipid bilayer of membranes with consequent effects on membrane components such as NADPH oxidase [70], an oxidase insensitive to KCN that can generate ROS. The direct interaction of pallidol with membranes may also be involved in the increase in root ionic permeability induced by EAF. There are reports of cytotoxic effects of pallidol on other cell types. Pallidol has antiproliferative activity against Caco-2 cells, with an IC_50_ of 306 ± 80 µg mL^−1^ [41], and is also active against some mycotoxigenic fungi when tested in vitro at a concentration of 100 µg mL^−1^ [71].

Plant metabolism changes substantially in the juvenile vegetative stage of seedling development (30-day-old seedlings) when the first eophylls develop and photosynthesis starts to generate metabolic energy in the form of ATP and NADPH and nutrients for the whole plant. In this phase, EAF exerts a double effect: stimulating growth at lower concentrations and inhibiting it at higher concentrations. It is likely that the active substance present in EAF affects distinct physiological processes with different sensitivities, with inhibitory action predominating over stimulatory action at higher concentrations.

The assessment of photosynthetic efficiency determined whether the effects of EAF on the growth of *I. grandifolia* seedlings were beneficial or not. EAF at a concentration of 250 µg mL^−1^ caused a reduction in substomatal carbon concentration (Ci), an effect unrelated to the change in stomatal conductance (g_s_) or transpiration (E), but associated with a marked increase in net CO_2_ assimilation (A), water use efficiency (WUE), and rate of immediate carboxylation of the photosynthetic system. Taken together, these results indicate a higher photosynthetic efficiency in the leaves probably due to an increased capacity of the enzymatic system of mesophyll cells to fix CO_2_, which may include Rubisco activity. This increase in CO_2_ fixation seems to be closely related to an increase in the efficiency of the photosynthetic electron chain and photon capture by photosystem pigments [72], as supported by the greater intensity of the SPAD index. This increase in the photosynthetic efficiency of seedlings treated with 250 µg mL^−1^ EAF correlated with the increase in the biometric parameters of seedling growth.

Treatment with 500 µg mL^−1^ EAF increased the stomatal conductance (g_s_) and transpiration (E), suggesting an increase in gas exchange and CO_2_ availability in the mesophyll. In agreement, an increase in photosynthetic rates was corroborated by an increase in net CO_2_ assimilation (A), water use efficiency (WUE), immediate carboxylation of the photosynthetic system (A/Ci), and the SPAD index.

Pallidol may be directly involved in these beneficial effects on photosynthesis, as suggested by the work of He et al. [73]. The authors found that pallidol, despite being ineffective in scavenging superoxide anions and hydroxyl radicals, is a potent and selective singlet oxygen quencher in aqueous systems. Singlet oxygen is generated in the PSII reaction center by the interaction of molecular oxygen with the excited triplet state of chlorophyll. Singlet oxygen production is stimulated by high-luminosity illumination and is involved in the photoinhibition process [74]. Photo-oxidative stress is prevented by singlet oxygen quenchers such as beta-carotene, tocopherol, and plastoquinone. According to He et al. [73], pallidol is effective at very low concentrations (IC_50_ = 14.5 µM) and has a high rate constant (k*a* = 1.71 × 10^10^). It seems plausible to suggest that this action of pallidol is involved in the greater photosynthetic efficiency of seedlings grown in the presence of 250 and 500 µg mL^−1^ EAF.

In plants treated with the highest concentration of EAF (1000 µg mL^−1^), which caused a significant reduction in seedling growth, the pattern of the responses was the opposite of those found at the lower concentrations. The net CO_2_ assimilation (A) was greatly reduced, possibly as a consequence of reduction in gas exchange, since transpiration (E) and stomatal conductance (g_s_) were also reduced, especially when compared to values found in the presence of 250 and 500 µg mL^−1^.

The reduction of stomatal conductance under water stress conditions is a regulatory mechanism that increases water use efficiency, allowing the plant to assimilate CO_2_ with minimal water loss, which leads to greater water savings by plants and greater synthesis of photoassimilates [75]. However, in the case of stomatal reduction induced by 1000 µg mL^−1^ EAF, both the water use efficiency (WUE) and the immediate carboxylation of the photosynthetic system (A/Ci) were reduced, despite an increase in substomatal carbon (Ci) concentration, a combination of effects that suggests an impairment on the metabolic pathways of the mesophyll. This phenomenon may be a consequence of the reduced chlorophyll content, as indicated by the strong reduction in SPAD indexes. Indeed, chlorophyll degradation has been reported to be one of the mechanisms of phytotoxicity of several allelochemicals in weeds [76,77].

Evaluation of the activity of some antioxidant enzymes in the leaves revealed increased SOD activity in seedlings treated with 250 µg mL^−1^. The activation of SOD may be a response to increased ROS generation, including the singlet oxygen, in chloroplasts due to the activation of photosynthetic rates observed under this condition. This is consistent with our previous assumption that pallidol exerted a protective effect against oxidative stress at this low concentration of EAF. In contrast, at higher concentrations an inhibitory action of EAF on SOD activity appears to predominate, following the same pattern of responses in seedling development.

At higher concentrations of EAF, the cytotoxic effects of pallidol found in early-stage seedlings and other cell types [41,71] appear to prevail over its beneficial effect on 30-day-old seedlings as a singlet oxygen quencher, as the observed changes in fluorometric and gas exchange analysis indicate damage to the photosystems.

These results indicated that a disturbance in energy metabolism may contribute to the weed suppression by pearl millet reported in the field. It should be mentioned that in addition to pallidol other active substances present in the EAF and ButF of pearl millet, and those with lower water solubility, may also contribute to weed suppression, as they can be released into the soil during mulch decomposition [78]. Further studies are still needed to identify the chemical nature of all the active allelochemicals present in the extracts of pearl millet straw.

A limitation of our study is that the experiments were conducted under controlled laboratory conditions. Research is needed to validate our findings under field conditions by measuring the concentration, retention, and degradation of the active constituents of the extracts in the soil environment, as these factors determine their uptake rates by target species. It is also important to investigate the allelopathic activity of the extracts among different cultivars of crops with which pearl millet is used for crop management.

Although field studies have demonstrated the ability of pearl millet to suppress weeds, it is known that the concentrations of allelochemicals released by the plant into the soil environment are generally low, and that chemical synthesis for widespread use is often difficult and expensive. The use of plant extracts is an alternative to overcome this obstacle, since pearl millet is a plant that can be cultivated on a large scale. Plant extracts concentrate the natural allelochemicals in a process in which solvents can be reused, and the extracts can be freeze-dried and stored for long periods.

## 5. Conclusions

The results of this study revealed that the aerial parts of *P. glaucum* contain soluble chemicals that inhibit the development of weed seedlings by altering energy metabolism and inducing cellular oxidative damage. A major current challenge in weed management is the wide spread of weed biotypes resistant to most chemical classes of herbicides. The use of *P. glaucum* as an allelopathic cover crop or the application of its active extracts can eliminate or minimize the use of herbicides, thus reducing selection pressure on weed populations and ensuring minimal environmental damage.

## Data Availability

Data are contained within the article or Supplementary Materials.

## Supplementary Materials

The following supporting information can be downloaded at: https://www.mdpi.com/article/10.3390/plants14020222/s1, Figure S1: Flowchart of the preparation and fractionation of the crude methanol extract of dried straw of the aerial parts of pearl millet; Figure S2: ESI (-)-HRMS/MS spectrum of Carasiphenol C; Figure S3: ESI (-)-HRMS/MS spectrum of pallidol; Table S1: Mean germination time, germination speed index and speed of accumulated germination of *Ipomoea grandifolia* grown for 24, 48, 72 and 96 h treated with 0 (control), 500, 1000 and 2000 µg mL^−1^ of the ethyl acetate fraction (EAF) of *Pennisetum glaucum*; Table S2: Data of the annotated compounds in the ethyl acetate fraction of *Pennisetum glaucum* by UHPLC-HRMS/MS. References [41,79] are cited in the Supplementary Materials.

## Abbreviations

A	Photosynthetic rate
ADP	Adenosine diphosphate
AIA	Auxin indoleacetic acid
ANOVA	Analysis of variance
AOX	Alternative oxidase
APX	Ascorbate peroxidase
AS	Speed of accumulated germination
ATP	Adenosine triphosphate
ButF	Butanolic fraction
CAT	Catalase
Ci	Intercellular CO_2_ concentration
COX	Cytochrome oxidase
E	Transpiration
EAF	Ethyl acetate fraction
EDTA	Ethylene diamine tetraacetic acid
GR	Glutathione reductase
gs	Stomatal conductance
GSSG	Oxidized glutathione
HUEM	Herbarium of the State University of Maringa
HSD	Honestly significant difference
IRGA	Infrared gas analyzer
KCN	Potassium cyanide
MDA	Malondialdehyde
NBT	Nitro blue tetrazolium
NADPH	Nicotinamide adenine dinucleotide phosphate
POD	Peroxidase
PPFD	Photosynthetic photon flux
PVP	Polyvinylpyrrolidone
ROS	Reactive oxygen species
S	Speed of germination
S.E.	Standard error
SOD	Superoxide dismutase
TBA	Thiobarbituric acid
TBARS	Thiobarbiturate-reactive substance
TCA	Trichloroacetic acid
$\bar{t}$	Mean germination time
WUE	Water use efficiency
