# Supplementary material for: Pearl Millet Cover Crop Extract Inhibits the Development of the Weed Ipomoea grandifolia by Inducing Oxidative Stress in Primary Roots and Affecting Photosynthesis Efficiency
Source: Plants (Basel). 2025 Jan 15;14(2):222. doi: 10.3390/plants14020222 (PMC11769161; doi:10.3390/plants14020222)
Supplement: Supplementary file 1 [file plants-14-00222-s001.zip › plants-3372879-supplementary.pdf]

Supplementary Information:

Methods

Figure S1 - Flowchart of the preparation and fractionation of the crude methanol extract of dried straw of the aerial parts of pearl millet.

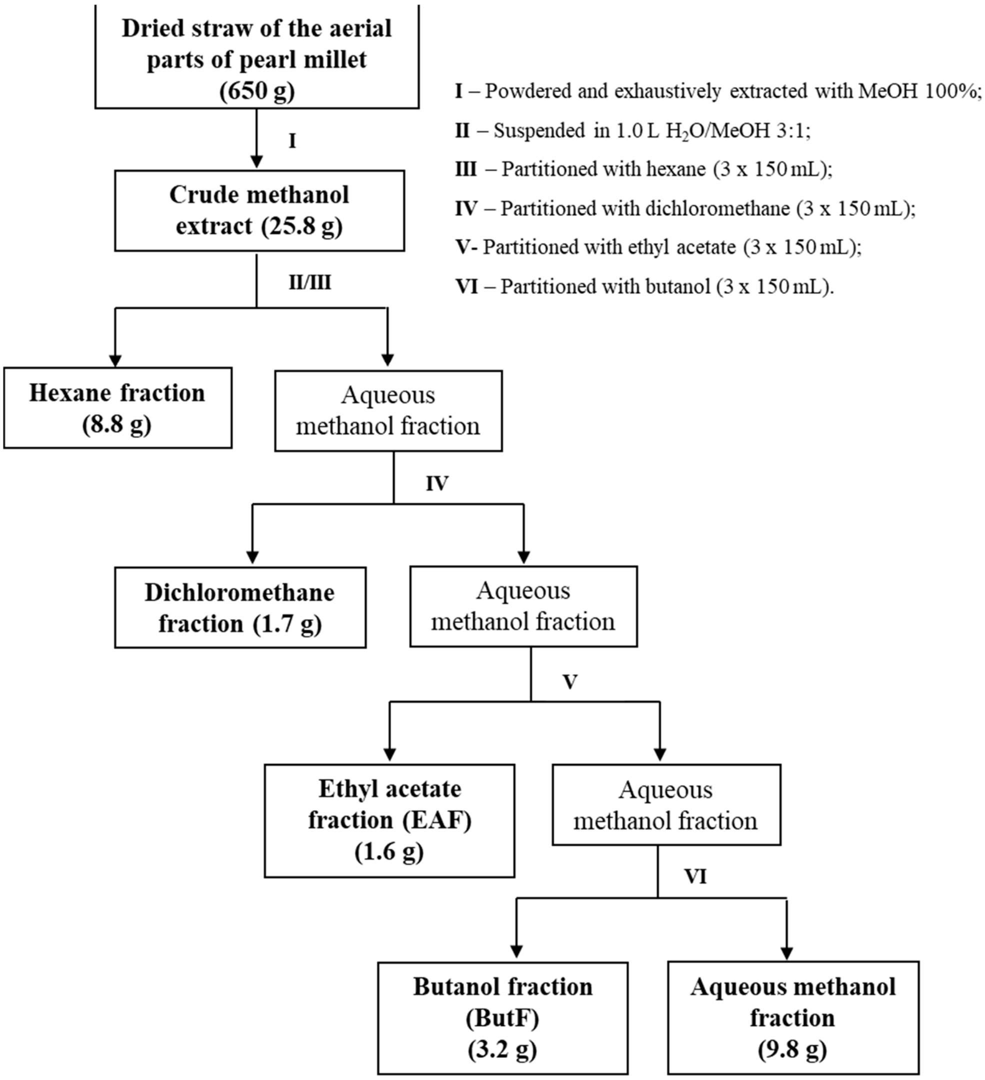

HPLC-DAD analysis

The EAF from the aerial parts of *Pennisetum glaucum* and pallidol standard were analyzed by high-performance liquid chromatography with diode array detector (Shimadzu, Prominence). A Supelcosil LC-18 (25 cm x 4.6 mm; 5 μm) column (Merck, Germany) was used for chromatographic separation. The samples were analyzed with a gradient elution program at the flow rate of 1.0 ml min^{-1}. The mobile phase was a mixture of acetonitrile (solvent A) and water (solvent B). The gradient elution program was: 5% A to 100% A (0–30 min) and 100% A (30–45 min) at a wavelength (λ) of 282 nm.

Analysis by UHPLC-HRMS/MS

The samples of EAF were prepared in MeOH (1.0 mg mL^{-1}) and chromatographic separations were performed using UHPLC on a Symmetry C18 column (75 × 2.0 mm i.d.; 1.6 μm Shim-pack XR-ODS III). The mobile phase consisted of 0.1% formic acid in H_2O (solvent A) and MeOH (solvent B). The gradient program was as follows: initial 0-1 min, using elution A-B (95:5, v/v), 1-3 min (30:70 v/v), 3-12 min (5:95 v/v) and kept at 95% B for 16 min at a flow rate of 0.2 mL min^{-1}. Injection volume was 3 μL. High resolution mass spectrometry analysis were carried out in a Q-TOF mass spectrometer via an electrospray ionization interface. The capillary voltage was operated in negative ionization mode, set at 4500 V, using sodium formate (10 μM) as calibrant. The dry gas parameters were set to 8 L min^{-1} at 200 ºC with a nebulization gas pressure of 4 bar. Collision-induced dissociation (CID) fragmentation was performed using argon (Ar) collision gas and collision energy from 0-30 eV. Spectra data of the investigated compounds were collected from *m/z* 50-1300 with a resolution of 50000, and with an acquisition rate of 5 spectrums per second. The ions of interest were selected by auto MS/MS scan fragmentation. The data processing software was Data analysis 4.3 (Bruker). Moreover, the mass error value was calculated.

Figures

Figure S2. ESI (-)-HRMS/MS spectrum of Carasiphenol C.

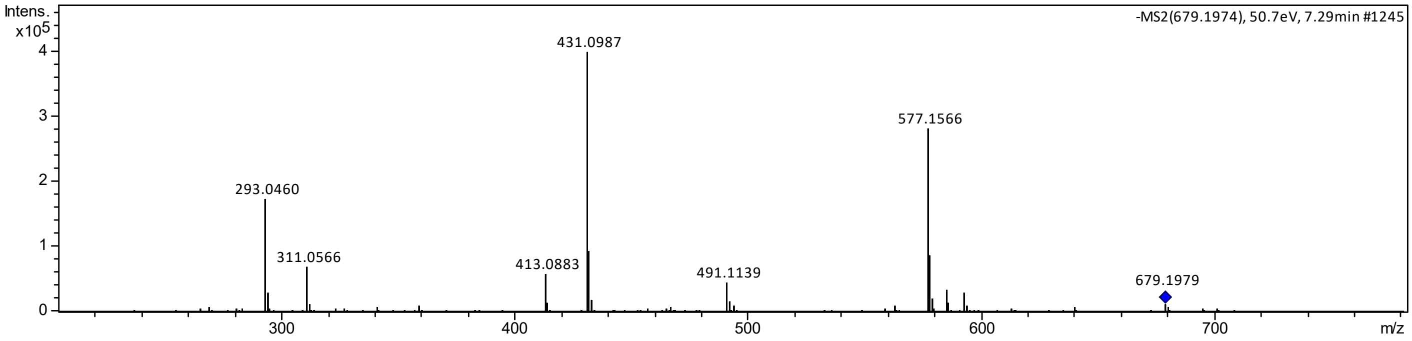

Figure S3. ESI (-)-HRMS/MS spectrum of pallidol.

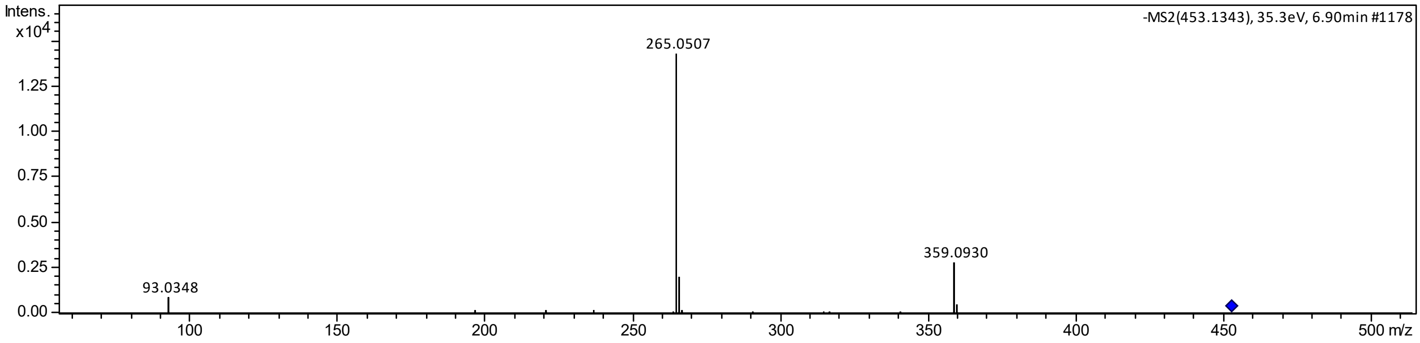

Tables:

Table S1. Mean germination time, germination speed index and speed of accumulated germination of *Ipomoea grandifolia* grown for 24, 48, 72 and 96 h treated with 0 (control), 500, 1000 and 2000 μg mL⁻¹ of the ethyl acetate fraction (EAF) of *Pennisetum glaucum*.

**EAF concentration (μg mL⁻¹)**	**Mean germination time ($\bar{t}$) (h)**	**Germination speed index (S)#**	**Speed of accumulated germination (AS)#**
0	24.19 ± 0.12 a	1.04 ± 0.00 a	4.32 ± 0.01 a
500	24.00 ± 0,00 a	1.01 ± 0.00 a	4.20 ± 0.03 b
1000	24.00 ± 0.00 a	1.01 ± 0.00 a	4.32 ± 0.02 a
2000	24.00 ± 0.10 a	1.03 ± 0.00 a	4.30 ± 0.03ab

#Seeds germinated per hour. Different letters in the same column indicate that means differed significantly according to Tukey's HSD test at $p \leq 0.05$ (n = 5).

Table S2. Data of the annotated compounds in the ethyl acetate fraction of *Pennisetum glaucum* by UHPLC-HRMS/MS.

	Molecular formula	Theoretical exact mass *m/z*	Experimental mass *m/z*	Mass error (ppm)	Rt (min)	Main MS/MS fragments in *m/z*
Pallidol[a]	$C_{28}H_{22}O_6$ [M - H]⁻	453.1344	453.1343	0.22	6.90	359; 265.
Carasiphenol C[b]	$C_{42}H_{32}O_9$ [M - H]⁻	679.1974	679.1974	0.0	7.29	577; 431; 293.

[a]Chen et al. [79]; [b]Silva et al. [41]; Rt: Retention time.
